# Breeding high-yielding drought-tolerant rice: genetic variations and conventional and molecular approaches

**DOI:** 10.1093/jxb/eru363

**Published:** 2014-09-09

**Authors:** Arvind Kumar, Shalabh Dixit, T. Ram, R. B. Yadaw, K. K. Mishra, N. P. Mandal

**Affiliations:** ^1^International Rice Research Institute (IRRI), DAPO Box 7777, Metro Manila, Philippines; ^2^Directorate of Rice Research (DRR), Rajendra Nagar, Hyderabad, India; ^3^National Rice Research Project (NRRP), Hardinath, Nepal; ^4^Central Rainfed Rice Research Station (CRURRS), Hazaribagh, Jharkhand, India

**Keywords:** Direct selection, drought, grain yield, marker-assisted breeding, QTLs, rice.

## Abstract

The increased occurrence and severity of drought stress have led to a high yield decline in rice in recent years in drought-affected areas. Drought research at the International Rice Research Institute (IRRI) over the past decade has concentrated on direct selection for grain yield under drought. This approach has led to the successful development and release of 17 high-yielding drought-tolerant rice varieties in South Asia, Southeast Asia, and Africa. In addition to this, 14 quantitative trait loci (QTLs) showing a large effect against high-yielding drought-susceptible popular varieties were identified using grain yield as a selection criterion. Six of these (*qDTY*_*1.1*_, *qDTY*_*2.2*_, *qDTY*_*3.1*_, *qDTY*_*3.2*_, *qDTY*_*6.1*_, and *qDTY*_*12.1*_) showed an effect against two or more high-yielding genetic backgrounds in both the lowland and upland ecosystem, indicating their usefulness in increasing the grain yield of rice under drought. The yield of popular rice varieties IR64 and Vandana has been successfully improved through a well-planned marker-assisted backcross breeding approach, and QTL introgression in several other popular varieties is in progress. The identification of large-effect QTLs for grain yield under drought and the higher yield increase under drought obtained through the use of these QTLs (which has not been reported in other cereals) indicate that rice, because of its continuous cultivation in two diverse ecosystems (upland, drought tolerant, and lowland, drought susceptible), has benefited from the existence of larger genetic variability than in other cereals. This can be successfully exploited using marker-assisted breeding.

## Summary

This study discusses improvement of popular rice varieties under drought through the identification and marker-assisted introgression of drought yield QTLs without any adverse effect on yield under normal conditions.

## Introduction

Around 90% of rice is grown and consumed in Asia. The semi-aquatic nature of rice and high water requirements for its cultivation make it much more prone to losses from drought than other cereals such as wheat and maize, which are better adapted to be grown with less water. As a result of the reduction in water availability and recent climate change scenarios, rice production is likely to be more severely affected by drought in Asia. Prior to the Green Revolution, traditional varieties adapted to the respective rice-growing ecosystems were cultivated across these areas. However, in the post-Green Revolution era, these varieties were replaced by a few fertilizer-responsive high-yielding varieties adapted to the irrigated ecosystem. These varieties were never screened for tolerance of drought and they suffer heavy yield losses even under mild stress conditions (Kumar *et al.*, 2008). On the other hand, the large and variable area under rice cultivation as well as different methods of rice cultivation (direct-seeded upland, transplanted lowland) make the crop unique in terms of its inherent variability available for tolerance of drought compared with other cereals.

Dry spells can occur at almost any time during the rice growth period in rain-fed areas, leading to drought stress of varying intensity. However, rice is highly sensitive to water stress at the reproductive stage (O’Toole 1982; Venuprasad *et al.*, 2007) as floral fertility in rice is extremely sensitive to water stress. Improving resilience to drought during floral development and anthesis is an important target (Richards *et al.*, 2010). This scenario has long been realized, and efforts have been made to understand the mechanisms related to drought tolerance as well as to develop varieties tolerant of drought. In the past, the major focus in breeding rice for drought tolerance was on secondary traits such as root architecture, water use efficiency, etc (Babu *et al.*, 2003; Lanceras *et al.*, 2004). It has also been believed that grain yield as a selection criterion is not suitable in breeding rice for drought tolerance. This has been attributed to the high complexity of genetic control of this trait, which leads to its low heritability under drought. Several experiments to standardize the procedures for uniform screening of segregating populations for grain yield under reproductive-stage drought (Venuprasad *et al*., 2007, 2008; Kumar *et al.*, 2008, 2009) showed moderate heritability of grain yield under drought, thereby confirming the suitability of grain yield as a selection criterion. It was also reported that, in large mapping populations, the correlation between high yield potential and good yield under drought was low but always positive (Kumar *et al.*, 2008), suggesting the possibility to combine high yield potential and good yield under drought successfully.

Once screening protocols were standardized, large-scale conventional breeding and quantitative trait locus (QTL) identification programmes were started, using yield as a selection criterion. This manuscript reports on the progress achieved in developing drought-tolerant varieties through new conventional breeding approaches based on direct selection for grain yield under drought, the identification of large-effect QTLs for grain yield under drought, and marker-assisted breeding using the identified QTLs to improve the grain yield of popular high-yielding but drought-susceptible varieties under drought.

## Materials and methods

### Donors, recipients, and segregating populations

Before the initiation of any breeding programme or mapping experiments, drought-tolerant donors were identified through screening of germplasm material. A majority of this material involved traditional *Aus*, *Indica*, and Basmati accessions. These accessions were evaluated for drought tolerance along with popular high-yielding varieties such as IR64, Swarna, and Sambha Mahsuri as checks, using grain yield as the selection criterion. Drought-tolerant lines identified through these experiments were evaluated for rice blast disease (caused by *Magnaporthe oryzae*) to identify lines tolerant of both drought and blast. These tolerant lines were crossed to popular high-yielding varieties to develop segregating populations for conventional breeding programmes and for developing mapping populations. Three main kinds of mapping populations were commonly used for the identification of QTLs for grain yield under drought. The first of these were recombinant inbred lines (RILs) developed by crossing two parents contrasting for the trait of interest followed by subsequent selfing and advancement through the single seed descent (SSD) method to achieve nearly homozygous lines (Kumar *et al.*, 2013). Backcross inbred lines (BILs) were developed through backcrossing (*n*=1–3) followed by self-pollination through the SSD method to develop BC_n_F_3:4_ populations. Advanced backcross (AB) populations proved to be specifically advantageous as they allowed the identification of lines with high yield potential and good plant and grain type because of the high percentage of the recipient parent. These could be used directly for testing and release in the target environment or could be used as parents for further backcross programmes to develop near-isogenic lines (NILs) of the recipient parent.

### Phenotyping of donors and mapping and segregating populations

#### Experimental designs and crop maintenance 

Screening of donors and mapping and segregating populations was conducted under upland and/or lowland reproductive-stage drought stress (RS) and irrigated non-stress (NS) conditions in dry season (DS) experiments at the International Rice Research Institute (IRRI). The experiments were planted in an α-lattice design with two replications in single or two-row plots with 5 m row length in lowland and 2.0–3.0 m row length in upland. Lowland experiments were carried out under transplanted conditions in which 21-day-old seedlings were transplanted in the field with a single seedling per hill. However, upland experiments were dry direct seeded. The row-to-row and plant-to-plant spacing of 20 cm×20cm in lowland and 25 cm×25cm in upland was maintained. Nitrogen, phosphorus, and potassium (NPK) were applied at the rate of 120:30:30 and 100:40:40kg ha^–1^ in lowland and upland, respectively. P and K were applied as basal, and N was applied in three splits, the first as basal, the second at maximum tillering, and the third at panicle initiation, in both lowland and upland. In order to control snails, Bayluscide (niclosamide, 0.25kg a.i. ha^–1^) was sprayed just after transplanting. At 4 days after transplanting (DAT; based on medium-duration lines), Sofit (pretilachlor±safener, 0.3kg a.i. ha^–1^) was sprayed to control weeds, followed by Furadan (carbofuran, 1kg a.i. ha^–1^) at 5 DAT and Cymbush (cypermethrin, 1 litre ha^–1^)±Dimotrin (cartap hydrochloride, 0.25kg a.i. ha^–1^) at 16 DAT to control insect pests.

#### Drought screening in upland conditions 

**Fig. 1.**
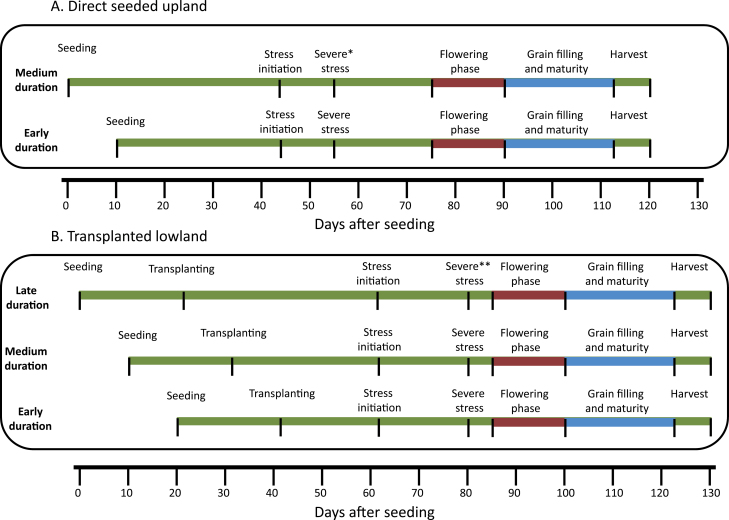
Protocol used for screening under reproductive-stage drought: (A) direct-seeded upland conditions and (B) transplanted lowland conditions. *Upland direct seeded: irrigated when the soil tensiometer shows a reading of –70 kPa at 30cm depth at 10:00h. **Lowland transplanted: irrigated when the water table depth recorded was below 90cm and susceptible checks showed severe leaf rolling and a leaf rolling score of 9 at 10:00h. (This figure is available in colour at *JXB* online.)

In upland conditions, donors and mapping populations were screened in sprinkler-irrigated dry direct-seeded trials. Up to 45 days after sowing (DAS; based on medium-duration lines), the trials were irrigated by sprinkler twice a week during establishment and early vegetative growth (Fig. 1A). Stress was initiated after this period by withholding irrigation, and plots were irrigated only when the soil water tension fell below –50 kPa at 30cm soil depth. At this soil water potential, most lines wilted and exhibited leaf drying. This type of cyclic stress is considered to be efficient in screening for drought tolerance in populations consisting of genotypes with a broad range of growth duration (Lafitte *et al.*, 2004) and it ensures that all lines receive adequate stress during reproductive development. Upland non-stress trials received the same cultural practices as the stress trials, except that irrigation was continued twice a week up to 10 d before harvest.

#### Drought screening in lowland conditions 

In lowland conditions, transplanted experiments were drained at 30 DAT and irrigation was withheld to impose drought stress at the reproductive stage (Fig. 1B). Stress was continued until severe leaf rolling (LR) was observed in at least 75% of the population lines and water table depth remained below 100cm for >2 weeks. Life-saving irrigation was provided thereafter through flash flooding, and water was drained after 24h to impose a second cycle of drought stress. Water table depth was measured by inserting a 1.1 m polyvinyl chloride (PVC) pipe in the experimental fields at regular intervals. Pipes were inserted to 1.0 m depth and 10cm of pipe remained above the soil surface. Depletion in the water table was measured through a meter scale daily after the onset of the stress.

### Data recorded

Data for days to 50% flowering (DTF), plant height (PHT), and grain yield (GY) were recorded. DTF was recorded when 50% of the panicles of the plants of each plot were exerted. PHT (cm) was measured at maturity from the soil surface to the tip of the panicle on the main tiller from three random plants of each plot and then the mean was calculated. Harvesting for GY was done at physiological maturity. Samples were harvested and dried to 12% moisture before weighing, and weights were converted to kg ha^–1^.

### Genotyping approaches, and QTL identification and validation

In the drought grain yield QTL identification as well as introgression programme, rice microsatellite [simple sequence repeat (SSR)] markers were widely used (Bernier *et al.*, 2007; Venuprasad *et al.*, 2009; Vikram *et al.*, 2011; Dixit *et al.*, 2012*a*; Mishra *et al.*, 2013; Yadaw *et al.*, 2013) for their easy PCR amplification and electrophoresis (Kumar *et al.*, 2013), the abundance of these markers across the genome that allows elaborate coverage, and their co-dominance nature that allows the detection of heterozygotes, making them suitable for genotyping all kinds of mapping populations. Whole-genome genotyping (WGG), selective genotyping (SG), and bulk segregant analysis (BSA) were used in different studies to identify QTLs for grain yield under drought. Each of these approaches has its own advantages and disadvantages. The selection of an approach was based on the type of mapping population and the aim of the study. The WGG approach was used in some QTL mapping studies (Vikram *et al.*, 2011). A full population was genotyped in this approach, with polymorphic markers spread evenly across the genome. Although the approach allowed the identification of major- and minor-effect QTLs as well as interaction between different loci, it was a relatively expensive and time-consuming approach. BSA (Michelmore *et al.*, 1991) that involved DNA pooling of lines based on the phenotypic extremes for developing high- and low-yielding bulks was used in several studies (Venupradsad *et al*., 2009; Vikram *et al.*, 2011; Ghimire *et al.*, 2012; Mishra *et al.*, 2013; Yadaw *et al.*, 2013; Dixit *et al.* 2014). These bulks were genotyped along with the parents with all polymorphic markers. The markers having bulk bands corresponding clearly to the parents were considered as candidates for full population genotyping and subsequent QTL analysis. BSA has proven to be a cost-effective approach although it does not allow the identification of minor QTLs and interaction between loci. Selective genotyping (Lebowitz *et al.*, 1987) that combines the advantages of both WGG and BSA but has some limitations was also used in some studies (Bernier *et al.*, 2007). A subset of the mapping population constituting 12% of the lines from the phenotypic extremes was selected for genotyping in this study. QTLs identified in different populations were evaluated at the IRRI across seasons for testing the consistency of effect. Whole populations or their subsets were evaluated in the target environment to test the effect of the QTLs.

### QTL identification

Linkage maps for the populations were developed using MapManager QTX (Manly *et al.*, 2001). To detect the relationship between markers and trait value, QTL cartographer 2.5.009 (Churchill and Doerge, 1994), Q gene 4.3.10 (Joehanes and Nelson, 2008), and QTLNetwork 2.1 (Yang *et al.*, 2008) were used. Single marker analysis (SMA) followed by composite interval mapping (CIM) were conducted to identify significant QTLs. All three software programs allow permutation tests to determine the significance threshold to identify significant QTLs. Some 500–1000 permutation tests were conducted to determine the threshold level. QTL cartographer and Q gene provided the LOD score to describe the significance of a QTL, while QTLNetwork used mixed model analysis for detecting QTLs and the significance of the QTLs was provided in terms of *F*-value.

### Introgression of QTLs

**Fig. 2.**
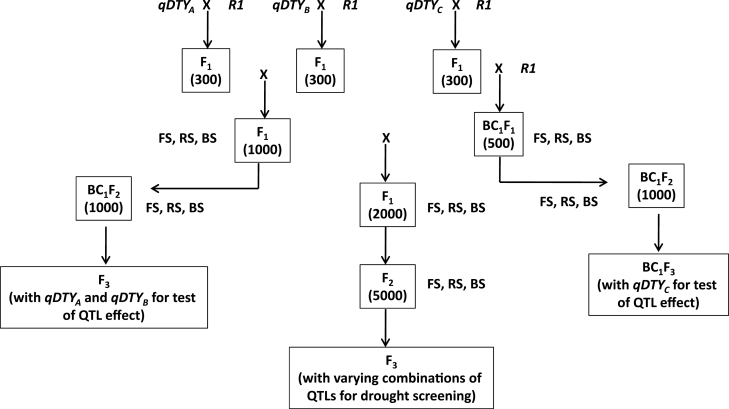
Marker-assisted backcrossing strategy used to develop NILs of a high-yielding popular variety with three *DTY* QTLs from different sources (from Kumar A, Dixit S, Henry A. 2013. Marker-assisted introgression of major QTLs for grain yield under drought in rice. In: Varshney RK, Tuberosa R, eds. *Translational genomics for crop breeding: abiotic stress, yield and quality*, Vol. 2. ©2013 John Wiley and Sons, Inc. with permission).

Efforts were made to identify at least three major QTLs in the background of popular high-yielding varieties from Asia. The initial introgression approach was to pyramid 2–3 QTLs in the background of a popular high-yielding variety for which they had been identified to obtain an economic yield advantage of 1.0–1.2 t ha^–1^ under drought in farmers’ fields. As many of the identified QTL regions were not fine mapped, all markers within the identified regions were used in the genotyping of the segregating generations to avoid a loss of candidate genes governing drought tolerance in the region due to crossover. To achieve this with reduced cost and minimum efforts, the sequential genotyping approach was followed. The backcrossed F_2_ segregants were first genotyped with a peak marker for each QTL, and lines possessing the donor allele at the peak marker were genotyped with flanking markers, followed by all markers within the QTL region. Further, because loci governing early days to flowering, plant height, and reduced yield under irrigated conditions were also detected within some of these QTL regions, larger segregating populations were used to break such linkages and develop dwarf high-yielding introgressed plants with DTF similar to those of recipient parents. A QTL pyramiding plan for pyramiding three QTLs coming from different sources in a variety is presented in Fig. 2. The use of large backcross populations for the identification of QTLs for grain yield under drought has proven advantageous for speeding up marker-assisted selection (MAS) and the quick recovery of the recipient genome in the background. With this technique, QTL mapping studies can be conducted simultaneously in three or more backcross populations derived from the cross of a common popular recipient with different drought-tolerant donors. QTLs can be identified through the use of genotyping techniques such as BSA. Once identified, lines with QTLs and the highest phenotypic similarity to the recipient parent can be selected and intercrossed. In each intercross F_1_ generation, foreground selection can be practised to select for F_1_ plants segregating for the respective QTLs. The final set of F_1_ plants segregating for all QTLs can be selfed to develop a large F_2_ population segregating for all QTLs. Plants fixed for different combinations of QTLs can then be identified from this population of F_2_ plants and advanced to the F_3_ generation. F_3_ lines so identified can be tested under drought stress and non-stress conditions. In a majority of the cases, single plant selection has to be made in the F_3_ generation to develop pure lines. Screening under drought stress conditions allows selection of the drought-tolerant plants within each line. Selection for plant type and grain type can also be practised in these lines to achieve the maximum possible similarity to the recipient parent as well as to identify plants better than the recipient parent. For the success of a QTL pyramiding programme, the nature of interactions between different QTLs introgressed and pyramided needs to be known. In the absence of this information, efforts can be made to develop NILs with all possible combinations of the target QTLs.

### Evaluation of introgressed lines

Selected NILs were screened under reproductive-stage drought (RS) and irrigated non-stress (NS) conditions. The introgressed lines along with the recipient high-yielding variety and drought-tolerant donors were evaluated first at IRRI for yield under RS and NS conditions. This was followed by evaluation of the selected lines for diseases—blast (caused by *M. oryzae*) and bacterial blight (caused by *Xanthomonas oryzae* pv*. oryzae*)—and grain and cooking quality traits. Selected lines from evaluation at IRRI for all these traits were tested under national/state trials in different countries. IR64 introgressed lines with *qDTY*_*2.2*_ and *qDTY*_*4.1*_—IR87707-445-B-B-B and IR87707-446-B-B-B—together with IR64 as a check were evaluated together at 51 locations in 2011 and 2012 under the All India Coordinated Rice Improvement Program in India and under the drought breeding programme in Nepal. IR84984-83-15-481-B, a *qDTY*_*12.1*_ NIL in the Vandana background, along with Vandana as a check were evaluated in 12 experiments conducted at the IRRI and at three locations in India in 2011–2012.

## Results and Discussion

### Identification and use of donors in QTL mapping and breeding programmes

The evolution of rice cultivars in drought-prone rainfed areas has allowed the development of a large number of landraces that possess high drought tolerance. However, a majority of these donors are landraces with low yield potential, low tillering, tall plant height, and poor grain and eating quality. Despite being known to possess drought tolerance, very few of them have been systematically characterized for the trait. A list of some of the donors characterized under reproductive-stage drought is presented in Table 1. Improved donors with good grain type (medium to long slender) and improved plant type (medium height, higher tillering, and lodging resistance) and tolerance of blast, such as Basmati 370, PSBRc 80, Aus 257, IR77298-14-1-2, IR83614-1002-B-B, and IR83614-1005-B-B were selected for direct use in the breeding programme. Some of these donors (Basmati 370 N22, Kali Aus, Dular and Apo) were also used in the QTL identification studies.

**Table 1.** Grain yield of drought-tolerant donors identified at the IRRI for use in conventional breeding and QTL mapping studies as compared with high-yielding susceptible varieties under reproductive-stage drought

Designation	Parentage	PHT	DTF	GY	BS	Suitability for use
Basmati 370	Traditional	113	32	5041	5	Conventional breeding
CT9993-5-10-1-M	CT 6241-2-2-1-3//Maravilha	96	28	3686	6	QTL mapping and pre-breeding
PSBRc 82	IR47761-27-1-3-6/IRRI 108	96	31	4630	6	QTL mapping and pre-breeding
PSBRc 68	IR43581-57-3-3-6/IR26940-20-3-3-3-1/Khao Dawk Mali 105	88	27	4433	7	QTL mapping and pre-breeding
PSBRc 80	IR50401-77-2-1-3/IR42068-22-3-3-1-3	81	34	4189	0	Conventional breeding
Aus Bak Tulsi	Traditional	89	84	4995	6	QTL mapping and pre-breeding
Kalia	Traditional	77	90	4752	3	QTL mapping and pre-breeding
Lal Aus	Traditional	86	97	4510	8	QTL mapping and pre-breeding
IR83614-1007-B-B	IR78875-131-B-1-2/IR64	79	86	4442	7	QTL mapping and pre-breeding
Aus 257	Traditional	96	89	4185	0	Conventional breeding
Kali Aus	Traditional	67	85	4032	7	QTL mapping and pre-breeding
IR77298-14-1-2	IR64 (WH)/Aday sel//3*IR64	65	91	4028	5	Conventional breeding
Dular	Traditional	82	88	3980	4	Conventional breeding and QTL mapping
IR83614-1002-B-B	IR78875-131-B-1-2/IR64	87	85	3179	4	Conventional breeding
IR83614-1005-B-B	IR78875-131-B-1-2/IR64	83	87	3155	5	Conventional breeding
IR57514-PMI-5-B-1-2	IR43581-57-3-3-6/Khao Dawk Mali 105//IR21836-90-3	96	90	2656	6	QTL mapping and pre-breeding
N22	Traditional	63	76	2249	0	Conventional breeding and QTL mapping
Apo	UPL RI 5/IR12979-24-1 (Brown)	66	100	1722	3	Conventional breeding and QTL mapping
IR36	IR1561-228-1-2/IR 1737//CR94-13	48	92	1331		Susceptible check
IR64	IR5657-33-2-1/IR2061-465-1-5-5	63	98	1054	0	Susceptible check
Sambha Mahsuri	RP 5/Mahsuri	72	30	754		Susceptible check
Swarna	Vasistha/Mahsuri	73	29	1073		Susceptible check
LSD_0.05_		5.24	12.2	643		

PHT, plant height; DTF, days to 50% flowering; GY, grain yield (kg ha^–1^); BS, blast score (on a scale of 0–9, where 0=highly tolerant and 9=highly susceptible.

Most of the traditional drought-tolerant donors are not used directly in breeding because of several undesirable traits that they possess. Table 2 compares drought-tolerant traditional donors and improved high-yielding drought-susceptible varieties in relation to morphology, phenology, yield, and growth-related traits. The specific differences in these characters led to the adaptation of these sets of lines in their specific environments (Supplementary Fig. S1 available at *JXB* online). For example, a majority of the drought-tolerant landraces show early flowering, tall plant height, low tillering, and low yield compared with medium to late flowering, semi-dwarf plant height, high tillering, and high yield of the high-yielding popular varieties. Some of these landraces have also been known to possess deep roots up to 70cm below the soil surface. Greater root length density at depth has also been reported in drought-tolerant genotypes such as Dular, Azucena, and Rayada compared with high-yielding drought-susceptible varieties such as IR64 (Henry *et al.*, 2011).

**Table 2.** Major morphological and phenological differences between traditional drought-tolerant donors and modern high-yielding varieties

Traits	Donors	Recipients
Lines	Landraces, improved tolerant varieties	High-yielding varieties
Yield potential	Low-medium (1.5–4.0 t ha^–1^)	Medium-high (>4 t ha^–1^)
Yield under drought	Low-medium (1.5–3.5 t ha^–1^)	Low (0–1.5 t ha^–1^)
Duration	Early-medium (DTF=60–85 d)	Medium-late (DTF=85–100 d)
Plant height	Semi-dwarf-tall	Dwarf
Tillering	Low-medium	High
Panicle length	Short-medium	Long
Root system	Shallow-deep rooted	Shallow rooted
Grain quality	Poor (landraces), good (improved donors)	Good (preferred)
Examples	N22, Moroberekan, Aus 276, Kali Aus	Swarna, IR64, TDK1, Sabitri

Donors with coarse grain type, tall plant height, and susceptibility to blast were used in the mapping study to identify QTLs and develop pre-breeding lines with improved tolerance of reproductive-stage drought and appropriate plant height and grain type for use in the breeding programme. Some of the pre-breeding lines that are widely used in a conventional breeding programme are mentioned in Table 3. These pre-breeding lines possessed high yield under non-stress conditions similar to the high-yielding varieties used as susceptible checks and good yield under reproductive-stage drought similar to the drought-tolerant checks (Table 3).

**Table 3.** Pre-breeding lines developed from mapping populations for use as improved donors in drought breeding programmes

Donor	QTL	GY (kg ha^–1^)	
		Non-stress	Stress
IR84984-83-15-185B	*qDTY*_*12.1*_	4933	2526
IR86918-B-92	*qDTY*_*1.1*_	4812	3831
IR86931-B-400	*qDTY*_*1.1*_	4910	3367
IR86929-B-482	*qDTY*_*1.1*_	5145	3517
IR86929-B-45	*qDTY*_*1.1*_	5230	3414
IR86929-B-320	*qDTY*_*1.1*_	5460	3185
IR86929-B-101	*qDTY*_*1.1*_	6728	3033
IR55419-04 (tolerant check)		3465	1732
IR77298-14-1-2-10 (tolerant check)		3036	1330
IR74371-54-1-1 (tolerant check)		5537	2705
IR74371-70-1-1 (tolerant check)		5327	2437
Apo (tolerant check)		5163	1661
MTU1010 (moderately susceptible check)		5451	624
Swarna (susceptible check)		3677	0
IR64 (susceptible check)		4511	250
LSD_0.05_		2566	2699

### Direct selection for grain yield under reproductive-stage drought

The suitability of grain yield as a selection criterion allowed the initiation of large-scale breeding programmes aimed at developing high-yielding drought-tolerant varieties with good grain quality and tolerance of major diseases. The conventional breeding programme at IRRI included screening of a large F_2_ population of ~5000 plants for rice blast. The selected tolerant plants were transplanted in the field and evaluated for reaction to bacterial blight at ~30 DAT by clipping the leaves with scissors infested with PXO61 and PXO86, two strains of *X. oryzae* pv. *oryzae*. Plants showing scores >3 based on the Standard Evaluation System (SES) were rejected. Single-plant selections were screened under reproductive-stage drought stress conditions in the F_3_ generation, and tolerant plants were selected based on grain yield. In F_4_ and F_5_ generations, selection was carried out in non-stress conditions with grain quality traits evaluated in the F_5_ generation. In the F_6_ generation, lines were screened under both reproductive-stage drought stress and non-stress conditions, and lines with high yield under both conditions were advanced to an observational yield trial (OYT). All trials until the F_6_ generation were conducted as unreplicated trials. In the OYT, lines were divided into groups based on crop duration, and lines belonging to each group were planted with checks in replicated yield trials in larger plots. Selected lines from OYTs constituted an advanced yield trial (AYT). These trials were conducted at IRRI and in the target environments in an α-lattice design with large plot sizes. Lines performing well in the target environment were advanced for release by the respective national systems. Combining high yield under reproductive-stage drought stress and non-stress in one genotype is the goal that breeders have been targeting for a long time. This is mainly because, in the years with well-distributed rainfall in these areas, the drought-tolerant varieties should provide high yield comparable with that of the popular high-yielding varieties. This cannot be achieved through direct cultivation of drought-tolerant landraces because of their low yield potential. The breeding programme described above allowed the development of several high-yielding drought-tolerant lines. Multilocation testing of these lines has led to the release of 17 varieties across South and Southeast Asia and Africa over the past 6–7 years (Table 4). Multilocation testing of elite breeding lines has also allowed a better understanding of the genotype×environment (G×E) interactions related to grain yield under reproductive-stage drought. In general, it has been observed that a majority of these lines perform best in their specific environments. This is also evident from the list of varieties presented in Table 5. A majority of these varieties were released in specific countries where they turned out to be the best performers. However, lines IR74371-70-1-1 and IR74371-54-1-1 were released under three different names in three countries—India, Bangladesh, and Nepal, and Nepal, the Philippines, and Nigeria, respectively—showing the stability of performance of these lines across environments. Regardless of the complexity of grain yield under reproductive-stage drought, lines selected under managed dry-season field experiments at IRRI were able to perform well in different countries. The success of this breeding programme points to the adaptability of lines across regions in shallow lowland environments of different countries not seen before, and this validates the earlier prediction that G×E interactions can be handled more accurately within the different topography (shallow lowland, medium lowland, or deep lowland) in the rainfed ecosystem (Kumar *et al.*, 2012).

**Table 4.** High-yielding drought-tolerant varieties developed from IRRI’s drought breeding programm e and released in different countries of South and Southeast Asia and Africa

Name	Designation	Days to maturity	Plant height (cm)	Country, release year, situation
Sahod Ulan 1	IR74371-54-1-1	110	104	Philippines 2009, RL, UP
Hardinath 1	IR80411-B-49-1-1	115	100	Nepal 2009, RL
Sahbhagi dhan	IR74371-70-1-1	110	104	India 2010, RL, UP
BRRI dhan56	IR74371-70-1-1	110	108	Bangladesh 2011, RL
Sookha dhan 3	IR74371-70-1-1	110	108	Nepal 2011, RL
Sookha dhan 1	IR74371-46-1-1	110	101	Nepal 2011, RL
Sookha dhan 2	IR74371-54-1-1	110	104	Nepal 2011, RL
Katihan 1	IR79913-B-176-B-4	105	90	Philippines 2011, UP
Sahod Ulan 3	IR81412-B-B-82-1	120	107	Philippines 2011, RL
Sahod Ulan 5	IR81023-B-116-1-2	115	130	Philippines 2011, RL
Sahod Ulan 6	IR72667-16-1-B-B-3	115	100	Philippines 2011, RL
Sahod Ulan 8	IR74963-262-5-1-3-3	125	100	Philippines 2011, RL
Inpago LIPI Go 1	IR79971-B-191-B-B	110	115	Indonesia 2011, UP
Inpago LIPI Go 2	IR79971-B-227-B-B	113	114	Indonesia 2011, UP
Sahod Ulan 12	IR81047-B-106-2-4	105	119	Philippines 2013, RL, DS
M’ZIVA	IR77080-B-B-34-3	120	130	Mozambique 2013, RL
UPIA3	IR74371-54-1-1	110	104	Nigeria 2013, RL

RL, rainfed lowland; UP, rainfed upland, DS, direct seeded.

**Table 5.** Major QTLs reported for high grain yield under upland and lowland reproductive-stage drought stress

QTL	Donor	Recipient	Ecosystem	Chr	Interval	*R* ^2^		Reported by
						P	G	
*qDTY*_*1.1*_	Dhagad deshi	Swarna	Lowland	1	RM431–RM104	32		Ghimire *et al.* (2012)
*qDTY*_*1.1*_	Dhagad deshi	IR64	Lowland	1	RM104–RM12091	9		Ghimire *et al.* (2012)
*qDTY*_*1.1*_	N22	Swarna	Lowland	1	RM11943–RM12091	13		Vikram *et al.* (2011)
*qDTY*_*1.1*_	N22	IR64	Lowland	1	RM11943–RM12091	17		Vikram *et al.* (2011)
*qDTY*_*1.1*_	N22	MTU1010	Lowland	1	RM11943–RM12091	13		Vikram *et al.* (2011)
*qDTY*_*1.1*_	Apo	IR64	Upland	1	RM486–RM472		58	Venuprasad *et al.* (2012*a* )
*qDTY*_*1.3*_	Kali Aus	IR64	Upland	1	RM488–RM315	5		Sandhu *et al.* (2014)
*qDTY*_*1.2*_	Kali Aus	MTU1010	Upland	1	RM259–RM315	7		Sandhu *et al.* (2014)
*qDTY*_*2.1*_	Apo	Swarna	Lowland	2	RM327–RM262		16	Venuprasad *et al.* (2009)
*qDTY*_*2.2*_	Aday Sel.	IR64	Lowland	2	RM236–RM279	11		Swamy *et al.* (2013)
*qDTY*_*2.2*_	Aday Sel.	IR64	Lowland	2	RM236–RM555	3		Swamy *et al.* (2013)
*qDTY*_*2.2*_	Aday Sel.	IR64	Lowland	2	RM236–RM555	9		Swamy *et al.* (2013)
*qDTY*_*2.2*_	Kali Aus	MTU1010	Upland	2	RM211–RM263	6		Sandhu *et al.* (2014)
*qDTY*_*2.2*_	Kali Aus	MTU1010	Lowland	2	RM211–233A	16		Palanog *et al.* (2014)
*qDTY*_*2.3*_	Kali Aus	IR64	Upland	2	RM263–RM573	6		Sandhu *et al.* (2014)
*qDTY*_*2.3*_	Kali Aus	IR64	Lowland	2	RM573–RM250	9		Palanog *et al.* (2014)
*qDTY*_*3.1*_	Apo	Swarna	Lowland	3	RM520–RM16030		31	Venuprasad *et al.* (2009)
*qDTY*_*3.1*_	IR55419-04	TDK1	Lowland	3	RM168–RM468	8		Dixit *et al.* (2014)
*qDTY*_*3.1*_	IR55419-04	TDK1	Upland	3	RM168–RM468	15		Dixit *et al.* (2014)
*qDTY*_*3.2*_	Aday Sel.	Sabitri	Lowland	3	RM569–RM517	23		Yadav *et al*. (2013)
*qDTY*_*3.2*_	N22	Swarna	Lowland	3	RM60–RM22	19		Vikram *et al.* (2011)
*qDTY*_*3.2*_	Moroberekan	Swarna	Lowland	3	id3000019–id3000946	8		Dixit *et al.* (unpublished)
*qDTY*_*3.2*_	Moroberekan	Swarna	Upland	3	id3000019–id3000946	19		Dixit *et al.* (unpublished)
*qDTY*_*4.1*_	Aday Sel.	IR64	Lowland	4	RM551–RM16368	11		Swamy *et al.* (2013)
*qDTY*_*6.1*_	Vandana	IR72	Upland	6	RM589–RM204		40	Venuprasad *et al.* (2012*b* )
*qDTY*_*6.1*_	Apo	IR72	Upland	6	RM589-RM204		63	Venuprasad *et al.* (2012*b* )
*qDTY*_*6.1*_	IR55419-04	TDK1	Lowland	6	RM586-RM217	9		Dixit *et al.* (2014)
*qDTY*_*6.1*_	IR55419-04	TDK1	Upland	6	RM586-RM217	36		Dixit *et al.* (2014)
*qDTY*_*6.2*_	IR55419-04	TDK1	Lowland	6	RM121-RM541	9		Dixit *et al.* (2014)
*qDTY*_*6.2*_	IR55419-04	TDK1	Upland	6	RM121-RM541	20		Dixit *et al.* (2014)
*qDTY*_*9.1*_	Aday Sel.	IR64	Lowland	9	RM105-RN434	13		Swamy *et al.* (2013)
*qDTY*_*9.1*_	Aday Sel.	IR77298-5-6-B-11	Lowland	9	RM105-RM434	19		Swamy *et al.* (2013)
*qDTY*_*10.1*_	MTU1010	N22	Lowland	10	RM216–RM304	5		Vikram *et al.* (2011)
*qDTY*_*10.2*_	Aday Sel.	IR64	Lowland	10	RM269–G2155	17		Swamy *et al.* (2013)
*qDTY*_*11.1*_	Moroberekan	Swarna	Upland	11	id11002304–id11006765	25		Dixit *et al.* (unpublished)
*qDTY*_*12.1*_	IR74371-46-1-1	Sabitri	Lowland	12	RM28166–RM28199	24		Mishra *et al.* (2013)
*qDTY*_*12.1*_	Way Rarem	Vandana	Upland	12	RM28048–RM28166	33	51	Bernier *et al.* (2007)

Modified from from Kumar A, Dixit S, Henry A. 2013. Marker-assisted introgression of major QTLs for grain yield under drought in rice. In: Varshney RK, Tuberosa R, eds. *Translational genomics for crop breeding: abiotic stress, yield and quality*, Vol. 2. ©2013 John Wiley and Sons, Inc. with permission.

### QTLs for high grain yield under reproductive-stage drought

The objective of any QTL identification programme should be the ultimate use of the identified QTLs for marker-assisted breeding. However, in the case of drought tolerance, the proportion of QTLs identified and used for MAS differs greatly. Although the term marker-assisted selection was first used in the literature more than two decades ago (Beckmann and Soller, 1986), very few studies have been able to use the identified QTLs for MAS and report them in publically available literature. A large proportion of QTL identification studies have targeted secondary traits related to drought tolerance (Babu *et al.*, 2003; Lanceras *et al.*, 2004). Some of the studies in the past have also focused on grain yield (Kumar *et al.*, 2007). However, very few studies have actually been able to use the identified QTLs for MAS.

In the case of rice, the choice of parents to develop mapping populations in a majority of these studies has been based on the trait of interest targeted for the study. For example, the selection of highly drought-susceptible parents adapted to the lowland ecosystem as recipient parents for a QTL identification study under direct-seeded upland conditions may allow the identification of large-effect QTLs but will definitely limit the future use of the identified QTLs. On the other hand, a desirable QTL allele with a large effect in a non-elite genetic background may not offer any improvement in the elite genetic background because the allele may already be ubiquitous in current varieties (Collins *et al.*, 2008).

The lack of repeatability of QTL effects across different populations—QTL×genetic background interaction (Q×G)—and across environments—QTL×environmental interaction (Q×E)—has been another factor limiting the use of QTLs in molecular breeding (Price *et al.*, 2002; Courtois *et al.*, 2003; Lafitte *et al.*, 2004; Bernier *et al.*, 2008). This demands that donor and recipient varieties be selected with appropriate consideration. The recipient variety for QTL studies should be an improved, high-yielding, drought-susceptible variety popular in the drought-prone environment. Due consideration for growth duration in addition to its drought tolerance, as well as resistance to insects and diseases, should be given when selecting a donor.

Using grain yield under reproductive-stage drought as a selection criterion, a number of large-effect QTLs for grain yield under reproductive-stage drought for both upland and lowland conditions have been identified. Table 5 presents a summary of such QTLs reported in rice. *qDTY*_*12.1*_ was the first reported large-effect QTL for grain yield under reproductive-stage drought (Bernier *et al.*, 2007). This QTL was identified in a population of 436 random F_3_-derived lines from a cross between upland rice cultivars Vandana and Way Rarem. Located between RM28048 and RM28166, this QTL explained an *R* ^2^ of 33% under severe upland reproductive-stage drought conditions. Later on, *qDTY*_*12.1*_ was also identified to show a similar high effect in lowland reproductive-stage drought in an IR74371-46-1-1/Sabitri population (Mishra *et al.*, 2013).

*qDTY*_*2.1*_ and *qDTY*_*3.1*_, two large-effect QTLs affecting grain yield under lowland reproductive-stage drought, were identified in a BIL population derived from a cross of high-yielding lowland rice variety Swarna and upland rice variety Apo. Both QTLs showed a very high effect under severe lowland reproductive-stage drought (*R* ^2^=16.3% and 30.7%). The effect of both these QTLs was also seen on other traits such as DTF and PHT. BSA was successfully used for the first time to identify such large-effect QTLs for grain yield under reproductive-stage drought (Venuprasad *et al.*, 2009). *qDTY*_*6.1*_, another large-effect QTL for grain yield under favourable aerobic and irrigated lowland conditions, was identified in this population (Venuprasad *et al.*, 2012*a*). This QTL explained an *R* ^2^ of up to 66% and 39%, respectively, under upland and lowland non-stress conditions.

A series of experiments began on F_3_-derived populations developed from the cross of drought-tolerant donor N22 with high-yielding mega-varieties Swarna, IR64, and MTU1010 that resulted in the identification of *qDTY*_*1.1*_, a large-effect QTL having an effect on grain yield under severe lowland reproductive-stage drought across these three populations. This QTL showed an *R* ^2^ of 13.4, 16.9, and 12.6% across two seasons of screening under severe lowland drought in N22/Swarna, N22/IR64, and N22/MTU1010 populations, respectively (Vikram *et al.*, 2011). QTLs for grain yield under reproductive-stage drought at this locus have also been reported in other populations derived from crosses of CT9993-5-10-1-M/IR62266-42-6-2 and Apo/IR64 (Kumar *et al.*, 2007; Venuprasad *et al.*, 2012*a*). In an IR64 background, four large-effect QTLs, *qDTY*_*2.2*_, *qDTY*_*4.1*_, *qDTY*_*9.1*_, and *qDTY*_*10.1*_, were identified in an Aday Sel/*4 IR64 BIL population (Swamy *et al.*, 2013). Similarly, *qDTY*_*3.2*_ was identified to show a large effect in an IR77298-14-1-2-10/Sabitri population (Yadaw *et al.*, 2013).

In terms of QTLs, *qDTY*_*1.1*_ showed an effect against three genetic backgrounds (Swarna, IR64,and MTU1010) from donor N22 (Vikram *et al.*, 2011) and against two genetic backgrounds (Swarna and IR64) from donor Dhagaddeshi (Ghimire *et al.*, 2012) in the lowland ecosystem and in the background of IR64 from donor Apo in the upland ecosystem (Venuprasad *et al.*, 2012*a*). *qDTY*_*3.1*_ showed an effect in lowland against Swarna (Venuprasad *et al.*, 2009) and BR11 (IRRI, unpublished) from donor Apo. *qDTY*_*2.2*_ showed an effect in IR64 from donor Aday Sel (Swamy *et al.*, 2013) in lowland and from Kali Aus in upland. *qDTY*_*3.2*_ showed an effect against Sabitri from donor IR77298-14-1-2 in lowland (Yadaw *et al.*, 2013) and in upland against Way Rarem from donor Vandana (Bernier *et al.*, 2007). *qDTY*_*6.1*_ showed an effect against Swarna in upland from donor Apo (Venuprasad *et al.*, 2012*b*) and in lowland against recipient variety TDK1 from donor IR55419-04 9 (Dixit *et al.*, 2014). *qDTY*_*12.1*_ showed an effect against Vandana from donor Way Rarem (Bernier *et al.*, 2007) in lowland and against recipient variety Sabitri in lowland from donor IR74371-46-1-1 (Mishra *et al.*, 2013).

In summary, the studies identified four QTLs (*qDTY*_*1.1*_, *qDTY*_*2.1*_, *qDTY*_*3.1*_, and *qDTY*_*6.1*_) to show an effect against Swarna, a popular variety in India, Nepal, and Bangladesh; six QTLs (*qDTY*_*1.1*_, *qDTY*_*2.2*_, *qDTY*_*3.2*_, *qDTY*_*4.1*_, *qDTY*_*9.1*_, and *qDTY*_*10.1*_) to show an effect in the background of IR64, a popular variety in many countries of South and Southeast Asia; two QTLs (*qDTY*_*3.2*_ and *qDTY*_*12.1*_) to show an effect against the background of Sabitri, a popular variety from Nepal; three QTLs (*qDTY*_*3.1*_, *qDTY*_*6.1*_, and *qDTY*_*6.2*_) to show an effect against TDK1, a popular variety from Laos; and one QTL (*qDTY*_*3.1*_) to show an effect against BR11, a popular variety from Bangladesh.

### QTL interactions with genetic background and environment

G×E interactions have always played a major role in the development of drought-tolerant crop varieties. The complexity of genetic control of these traits leads to large differences in the performance of lines across variable environments. However, for MAS to be worthwhile, it is important that the identified QTLs show large and consistent effects under varying environmental conditions and across a wide range of genetic backgrounds (Bernier *et al.*, 2009; Vikram *et al.*, 2011). It is therefore important that the QTLs have a genetic effect large enough to be effective across a variety of environmental conditions and drought intensities. One way to overcome this could be to choose a recipient parent suitable for the target environment to develop the mapping population and screen the mapping population in the target environment under naturally occurring drought stress conditions. However, the surety of achieving the desired level of drought stress in the field in the rainy season is much less. In such cases, it is often advantageous to screen mapping populations under managed drought stress conditions to identify QTLs and to validate the QTL effect by screening the full set or a subset of the mapping population in the target environment. Bernier *et al.* (2009), from 21 experiments conducted at the IRRI and in eastern India, confirmed that *qDTY*_*12.1*_ showed an increased effect with increasing severity of drought stress. Similarly, two large-effect QTLs (*qDTY*_*12.1*_ and *qDTY*_*3.2*_) identified in two different populations were validated for their effect in Nepal by phenotyping the full mapping population in Nepal in the second season (Mishra *et al.*, 2013; Yadaw *et al.*, 2013).

Another major limitation in the use of QTLs in MAS despite their large effects is their specificity to genetic backgrounds. It may be very advantageous if QTLs with large effects show an effect across multiple genetic backgrounds. QTL studies for grain yield under drought have allowed the identification of at least seven QTLs that have shown an effect across multiple genetic backgrounds: *qDTY*_*1.1*_, *qDTY*_*2.2*_, *qDTY*_*2.3*_, *qDTY*_*3.1*_, *qDTY*_*3.*2_, *qDTY*_*6.1*_, and *qDTY*_*12.1*_. Genotyping strategies such as BSA also make it possible to screen a large number of mapping populations simultaneously for the presence of a QTL affecting grain yield in more than one background. Table 5 summarizes the effect of QTLs identified in a particular genetic background from different donors under different ecosystems. It has been observed that the effect of the same QTL varies with donors and recipients, as well as with the environment in which it is detected. For example, one of the most consistent QTLs, *qDTY*_*1.1*_, contributed by donor N22, was identified in the background of mega-varieties MTU1010, IR64, and Swarna (Vikram *et al.*, 2011). This QTL was also contributed by another donor, Dhagaddeshi, to IR64 and Swarna (Ghimire *et al.*, 2012). In both studies, the QTL was identified using BSA.

The interaction of QTLs with genetic backgrounds has been a major limitation in the use of QTLs for MAS. Epistatic interactions play an important role in determining the level of effect of a QTL across genetic backgrounds. This phenomenon is also observed with *DTY* QTLs. The effect of genetic background can most clearly be observed in the case of *qDTY*_*12.1*_. Despite being one of the largest QTLs reported for grain yield under reproductive-stage drought, explaining 51% of the genetic variation, a study of epistatic interaction in a Vandana/Way Rarem population showed two loci (*qDTY*_*2.3*_ and *qDTY*_*3.2*_) to be interacting with *qDTY*_*12.1*_ and significantly enhancing the yield of *qDTY*_*12.1*_-positive lines (Dixit *et al.*, 2012*b*).

### Marker-assisted breeding with *DTY* QTLs and products developed

The rapid development of drought-tolerant versions of popular varieties can be one of the strategies to ensure rice production under reproductive-stage drought without compromising on yield potential and the preferences of farmers and consumers. Moreover, because of the low positive correlation between high yield potential and grain yield under reproductive-stage drought, marker-assisted breeding using well-defined QTLs allows precise combining of high yield potential and good yield under reproductive-stage drought. Apart from this, marker-assisted breeding also allows rapid product development with reduced efforts and with relatively smaller segregant populations. However, marker-assisted breeding for drought tolerance requires careful planning from the start of the QTL identification process.

Large-scale QTL identification and introgression programmes in different popular drought-susceptible varieties showed the specific compatibility of QTLs in terms of yield under reproductive-stage drought. Some of these large-effect QTLs showed an effect in a majority of the genetic backgrounds, stress severities, and ecosystems, while others showed more specificity for these factors. It therefore becomes important to characterize the compatibility of these QTLs in different genetic backgrounds and environments. For example, the combination of *qDTY*_*12.1*_ with *qDTY*_*2.3*_ and *qDTY*_*3.2*_ led to a higher yield advantage than in lines with *qDTY*_*12.1*_ alone under upland stress conditions in a Vandana/Way Rarem F_3_-derived population (Dixit *et al.*, 2012*b*). This combination of QTLs also led to an advantage under lowland stress conditions in which the effect of *qDTY*_*12.1*_ alone was not observed. Similarly, in an IR64 background, lines with *qDTY*_*2.2*_ and *qDTY*_*4.1*_ showed a higher yield advantage under reproductive-stage drought than lines with four QTLs (*qDTY*_*2.2*_, *qDTY*_*4.1*_, *qDTY*_*9.1*_, and *qDTY*_*10.1*_) under lowland stress conditions (Swamy *et al.*, 2013). The combination of *qDTY*_*1.1*_, *qDTY*_*2.1*_, and *qDTY*_*3.1*_ together has been found more advantageous than having one or two QTL combinations in a Swarna–Sub1 background. Efforts are being made to bring these QTLs together in one genetic background to understand their interactive effects on grain yield under reproductive-stage drought.

QTL identification studies at the IRRI identified a set of QTLs with large effects in the background of rice varieties Swarna, IR64, TDK1, Sabitri, and BR11, and enlisted the set of QTLs that should be used to improve varieties for grain yield under reproductive-stage drought in lowland (*qDTY*_*1.1*_, *qDTY*_*2.2*_, *qDTY*_*3.1*_, *qDTY*_*3.2*_, and *qDTY*_*12.1*_) and upland (*qDTY*_*2.3*_, *qDTY*_*3.2*_, and *qDTY*_*12.1*_). Although the set of identified QTLs mentioned above will bring about yield improvement under reproductive-stage drought in a majority of the high-yielding backgrounds, it is not necessary that they be the best combination of QTLs for every background. This is due to the interaction between the QTLs and the genetic background of the recipient varieties. In such cases, the development of a BC_1_F_3_ BIL population to identify the best QTL combination before proceeding further in the pyramiding programme could be an appropriate strategy.

In many cases, *DTY* QTLs link tightly to traits such as plant height and earliness. It becomes important to develop large BC_n_F_1_ (n being the number of backcrosses) populations in each cycle of backcrossing to allow enough recombination to break these linkages. It is also required that a large number of BC_n_F_2_ segregants with different QTL combinations be selected and precisely phenotyped under reproductive-stage drought as against the selection of fewer plants practised for traits with simpler genetic control. Proper drought phenotyping of different combinations of QTLs allows the selection of lines with a positive interaction between different QTLs and the genetic background of the recipient variety, allowing breeders to capture a high yield advantage under reproductive-stage drought.

Table 6 presents a list of varieties improved or being improved by the introgression of *DTY* QTLs at the IRRI. A marker-assisted breeding programme for seven popular varieties, Swarna, IR64, Vandana, Sabitri, TDK1, Anjali, and Sambha Mahsuri, was undertaken. For Swarna, a combination of three QTLs (*qDTY*_*1.1*_, *qDTY*_*2.1*_, and *qDTY*_*3.1*_) was pyramided along with *Sub1*, the large-effect QTL for tolerance of submergence. Lines tolerant of both drought and submergence are at the final stages for testing in the target environment. Similarly, IR64 introgression lines with *qDTY*_*2.2*_ and *qDTY*_*4.1*_ were developed and have been tested in a wide range of environments for tolerance of drought. Two upland rice varieties were also improved through marker-assisted introgression of *DTY* QTLs: Vandana and Anjali. *qDTY*_*12.1*_ was introgressed in Vandana, and *qDTY*_*12.1*_ and *qDTY*_*3.1*_ were introgressed in Anjali. Three large-effect QTLs (*qDTY*_*3.1*_, *qDTY*_*6.1*_, and *qDTY*_*6.2*_) were identified and introgressed in TDK1, a popular variety from Lao PDR. Similar to Swarna, these QTLs were pyramided along with *Sub1* to confer tolerance of both drought and submergence. Two other varieties, Sabitri and Sambha Mahsuri, that are popular in Nepal and in India, respectively, are also in the marker-assisted breeding pipeline. Sabitri is being introgressed with *qDTY*_*3.2*_ and *qDTY*_*12.1*_. The effect of these two QTLs in Nepal has already been validated in two separate QTL identification programmes (Mishra *et al.*, 2013; Yadaw *et al.*, 2013). The combination of *qDTY*_*2.2*_ and *qDTY*_*4.1*_ is being introgressed into Sambha Mahsuri. A large-scale QTL introgression programme is also underway to introgress six *DTY* QTLs from different sources along with *Sub1* in IR64.

**Table 6.** List of DTY QTLs pyramided in the background of popular rice varieties through marker-assisted breeding along with QTLs for tolerance of other stresses

Variety	Target ecosystem	*DTY* QTLs used	Other QTLs	Current stage
IR64	Rainfed lowland	*qDTY*_*2.2*_, *qDTY*_*4.1*_		Released in Nepal, identified for release in India, tested for release in Bangladesh
Swarna	Rainfed lowland	*qDTY*_*1.1*_, *qDTY*_*2.1*,_ *qDTY*_*3.1*_	*Sub1*	Testing and validation in progress
Vandana	Rainfed upland	*qDTY*_*12.1*_		Testing and validation in progress
Sabitri	Rainfed lowland	*qDTY*_*3.2*_, *qDTY*_*12.1*_		Introgression ongoing
Anjali	Rainfed upland	*qDTY*_*3.1*_, *qDTY*_*12.1*_		Testing and purification in progress
TDK1	Rainfed lowland	*qDTY*_*3.1*_, *qDTY*_*6.1*_, *qDTY*_*6.2*_	*Sub1*	Testing and purification in progress
Sambha Mahsuri	Rainfed lowland	*qDTY*_*2.2*_, *qDTY*_*4.1*_		Testing and purification in progress
IR64	Rainfed lowland	*qDTY*_*1.1*_, *qDTY*_*1.2*_, *qDTY*_*2.2*_, *qDTY*_*12.1*_, *qDTY*_*2.3*_, *qDTY*_*3.2*_	*Sub1*	Testing and purification in progress

### The performance of *DTY* QTL introgressed lines in South Asia

**Fig. 3.**
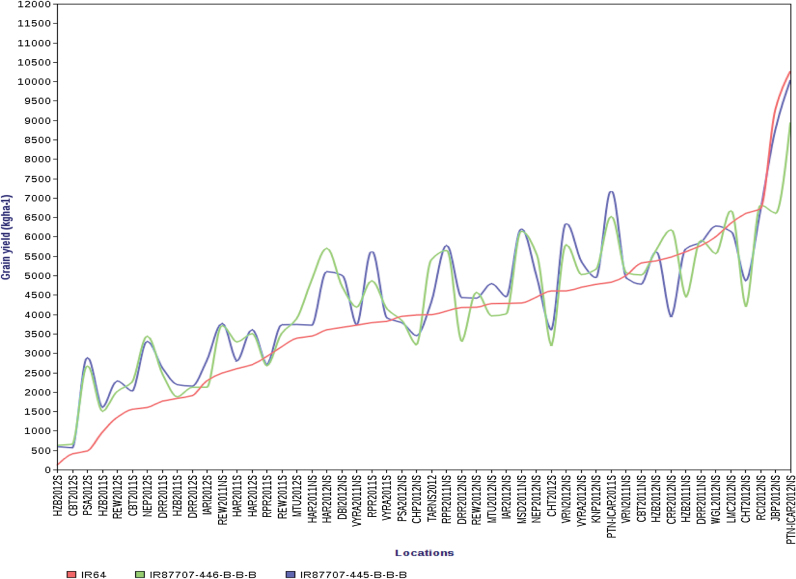
Performance of NILs IR87707-445-B-B-B and IR87707-446-B-B-B compared with IR64 in 51 experiments conducted under varying levels of drought stress and non-stress conditions across India and Nepal. Experiments are arranged in order of increasing mean yield of IR64, classifying HZB2012S–DRR2012S as severe stress experiments, IARI2012S–RPR2011S as moderate stress experiments, REW2011S–TARNS2012 as mild stress experiments, and RPR2011NS–PTN-ICAR2012NS as non-stress experiments. (This figure is available in colour at *JXB* online.)

**Fig. 4.**
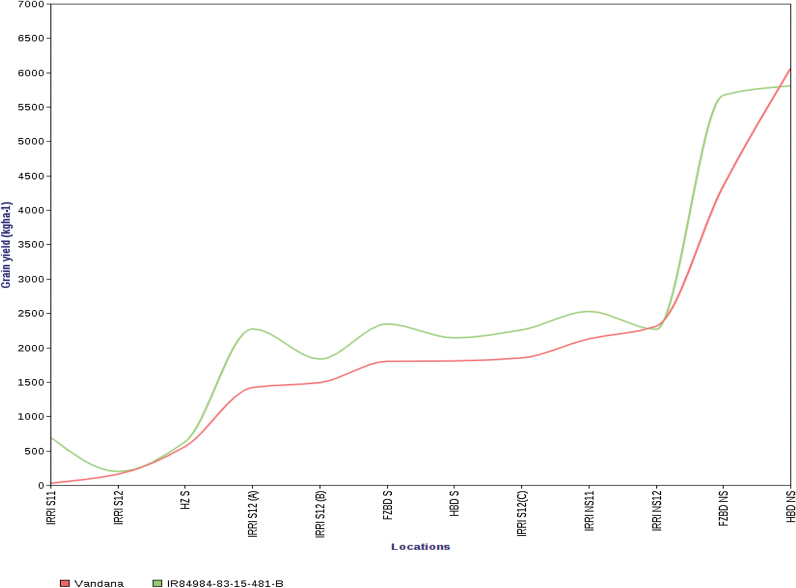
Performance of IR84984-83-51-481-B compared with Vandana in 12 experiments conducted under varying levels of drought stress and non-stress conditions at the IRRI and in India. Experiments are arranged in order of increasing mean yield of Vandana, classifying IRRIS11–HZS as severe stress experiments, IRRIS12(A)–IRRIS12(C) as moderate stress experiments, and IRRINS11–HBDNS as non-stress experiments. (This figure is available in colour at *JXB* online.)

**Fig. 5.**
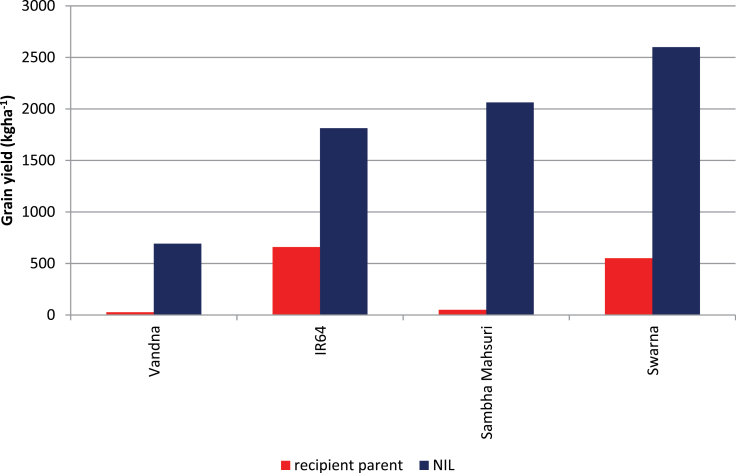
Yield gains under drought obtained in rice through introgression and pyramiding of QTLs with additive effects in high-yielding genetic backgrounds. (This figure is available in colour at *JXB* online.)

IR64 lines introgressed with *qDTY*_*2.2*_ and *qDTY*_*4.1*_ (IR87707-445-B-B-B and IR87707-446-B-B-B) were tested in 51 experiments conducted across India and Nepal. Both lines showed increased yield at most of the sites, where drought conditions varied from irrigated conditions with no drought to mild drought and moderate drought, to very severe drought (Fig. 3). IR87707-445-B-B-B has been identified for release in India and IR87707-446-B-B-B has been identified for release in Nepal (see mean and LSD_0.05_ details in Supplementary Table S1 at *JXB* online). Under severe reproductive-stage drought stress conditions in a rainout shelter as well as in the field, the IR64 NILs showed a yield advantage of 100–500 percentage points over the recurrent parent IR64 (Table 7). In a farmers’ preference score conducted in Nepal, the IR64 introgressed lines showed a higher preference score of +0.34 and +0.43 for IR87707-446-B-B-B and +0.15 and +0.27 for IR87707-445-B-B-B as against –0.03 and –0.05 for IR64 in 2011 and 2012, respectively. This is the first product developed through marker-assisted breeding of *DTY* QTLs released for commercial cultivation. A Vandana introgressed line with *qDTY*_*12.1*_, IR84984-83-15-481-B, outperformed Vandana under all trials in upland conditions and had a high yield similar to that of Vandana under lowland irrigated non-stress conditions (Fig. 4; Supplementary Table S2). The introgression of different combinations of *DTY* QTLs has also allowed researchers to understand the effect of specific combinations of the QTLs and the yield improvement achieved in different cultivars. For example, the introgression of one QTL in Vandana led to an increase in yield of 0.5 t ha^–1^ under drought, and the introgression of two QTLs led to an increase in yield of >1.0 t ha^–1^ in IR64 and Sambha Mahsuri, whereas the introgression of three QTLs in Swarna showed a yield advantage of >1.5 t ha^–1^ under drought (Fig. 5).

**Table 7.** Percentage yield advantage of IR64 NILs over recurrent parent IR64 under severe and moderate drought in rainout shelter and field conditions across eight locations in India in 2012

Location	Stress intensity	Percentage advantage over IR64	
		IR87707-445-B- B-B	IR87707- 446-B-B-B
Hazaribagh	Severe	396.6	362.2
Coimbatore	Severe	38.6	61.1
Pusa	Severe	500.0	433.3
Hyderabad	Severe	12.9	13.2
New Delhi	Moderate	22.6	2.4
Patna	Moderate	48.8	13.0
Rewa	Moderate	69.0	82.6
Maruteru	Moderate	10.4	3.9

### Concluding comments

Parallel cultivation of rice in two diverse ecosystems, upland and lowland, has allowed the evolution of the crop in two very diverse environments. On the one hand, some upland-adapted drought-tolerant rice varieties are characterized by traits such as early flowering and root systems suitable for dry conditions, whereas, on the other hand, some drought-susceptible lowland-adapted rice varieties are characterized by medium to late maturity, high input responsiveness, and specificity to anaerobic growing environments. The existence of such large diversity for drought tolerance puts rice in a unique position, with much higher genetic diversity available for drought tolerance. The presence of conserved regions conferring drought tolerance in upland rice and the high susceptibility of high-yielding post-Green Revolution varieties provide unique opportunities for plant breeders to move drought tolerance alleles from upland drought-tolerant donors to lowland drought-susceptible rice varieties. Swamy *et al.* (2011), through a study on a panel of random drought-tolerant donors for the identified drought yield QTLs, reported the presence of *qDTY*_*12.1*_ in 85% of the lines, followed by *qDTY*_*4.1*_ in 79% of the lines and *qDTY*_*1.1*_ in 64% of the lines, thus validating the high presence of these identified QTLs in drought-tolerant donors. Advances in molecular biology have provided new opportunities for breeders to identify such regions, refine these regions through fine mapping, and move those regions into drought-susceptible varieties, an opportunity that was not available a few years back to break the yield improvement barrier under drought.

## Supplementary data

Supplementary data are available at *JXB* online.

Figure S1. Difference in plant types and drought response of upland-adapted and lowland-adapted cultivars under severe drought.

Table S1. Mean grain yield (kg ha^–1^) of IR87707-445-B-B-B, IR87707-446-B-B-B, and IR64, and LSD_0.05_ values for experiments conducted across India and Nepal.

Table S2. Mean grain yield (kg ha^–1^) of IR84984-83-15-481-B and Vandana, and LSD_0.05_ values for experiments conducted across India and at IRRI.

Supplementary Data

## Abbreviations:

AB: advanced backcross
BIL: backcross inbred line
BSA: bulk segregant analysis
CIM: composite interval mapping
DAS: days after sowing
DAT: days after transplanting
DTF: days to 50% flowering;
GY: grain yield
NS: irrigated non-stress
LR: leaf rolling
MAS: marker-assisted selection
NIL: near-isogenic line
PHT: plant height
PVC: polyvinyl chloride
Q×E: QTL×environment
Q×G: QTL×genetic background
RIL: recombinant inbred line
RS: reproductive-stage drought stress
SG: selective genotyping
SIM: simple interval mapping
SMA: single marker analysis
SSD: single seed descent
WGG: whole-genome genotyping.
