# Supplementary material for: Breeding high-yielding drought-tolerant rice: genetic variations and conventional and molecular approaches
Source: J Exp Bot. 2014 Sep 9;65(21):6265–78. doi: 10.1093/jxb/eru363 (PMC4223988; doi:10.1093/jxb/eru363)
Supplement: Supplementary Data [file supp_65_21_6265__index.html]

Breeding high-yielding drought-tolerant rice: genetic variations and conventional and molecular approaches — Breeding high-yielding drought-tolerant rice: genetic variations and conventional and molecular approaches — Supplementary Data 

# Breeding high-yielding drought-tolerant rice: genetic variations and conventional and molecular approaches

## Supplementary Data

Data files

**Files in this Data Supplement:**

- Supplementary Data - Supplementary Data
- Supplementary Data - Supplementary Data
