# Supplementary material for: Breeding high-yielding drought-tolerant rice: genetic variations and conventional and molecular approaches
Source: J Exp Bot. 2014 Sep 9;65(21):6265–78. doi: 10.1093/jxb/eru363 (PMC4223988; doi:10.1093/jxb/eru363)

Breeding high-yielding drought-tolerant rice: genetic variations and conventional and molecular approaches

Arvind Kumar, Shalabh Dixit, T Ram, R B Yadaw, K K Mishra, and N P Mandal

Supplementary Data

Supplementary Tables S1-S2 – see separate file

Figure S1: Difference in plant types and drought response of upland-adapted and lowland-adapted cultivars under severe drought.

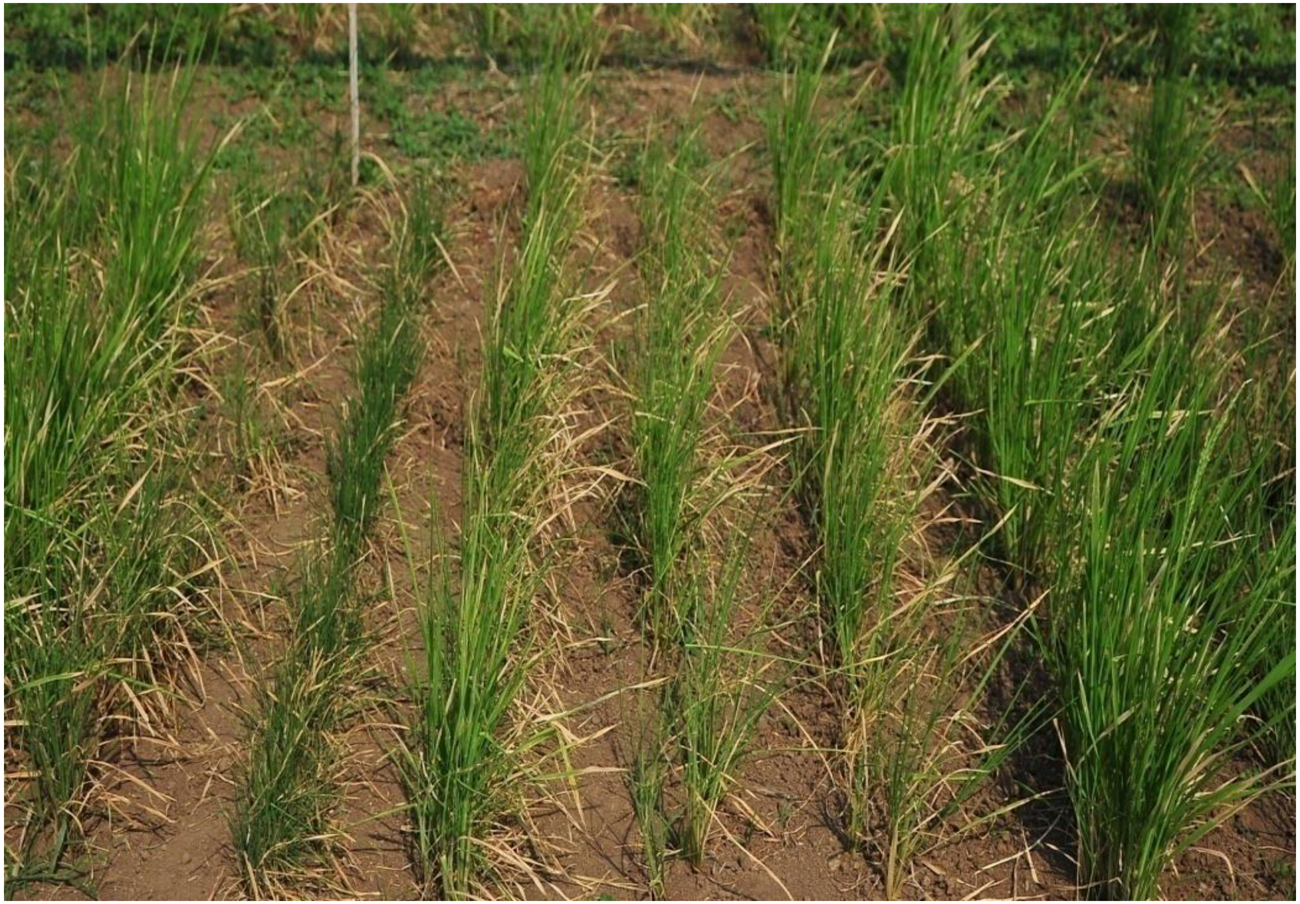

Supplement: Supplementary Data [file supp_eru363_jexbot120972_file002.pdf]
